# Niacin mitigates heat stress-induced reduction in performance of Taihe black-boned silky fowl through modulation of gut microorganisms and short-chain fatty acids

**DOI:** 10.3389/fvets.2025.1592101

**Published:** 2025-06-23

**Authors:** Wenliang Mei, Chuanbin Chen, Xiaona Gao, Wenyan Zhang, Ziyu Hu, Mingren Qu, Gen Wan, Lanjiao Xu

**Affiliations:** Jiangxi Key Laboratory of Animal Nutrition, Nanchang, China

**Keywords:** TBsf, nicotinic acid, heat stress, cecum microbiota, SCFA

## Abstract

This study aimed to evaluate the dose-dependent effects of nicotinic acid (NA) on growth performance, cecal short-chain fatty acid (SCFA) profiles, and gut microbiome composition in Taihe black-boned silky fowl (TBsf) under heat stress (HS) conditions. In the experiment, 150 healthy male TBsf were selected and randomly assigned to five treatment groups, with 30 individuals per group. The HS groups were fed a basal diet supplemented with 0, 200, 400, and 800 mg/kg of NA, respectively. HS significantly elevated body temperature and serum heat shock protein 70 (HSP70) concentration compared with the control group (thermal neutral, TN) (*p* < 0.05), while reducing the growth performance and apparent digestibility of crude protein in TBsf (*p* < 0.05). The addition of 800 mg/kg NA to the diet significantly reduced body temperature. Compared with the HS group, the incorporation of 200–800 mg/kg NA significantly decreased serum HSP70 levels, significantly increased the average daily gain (ADG) of TBsf, and significantly decreased the feed-to-gain ratio (F/G) (*p* < 0.05). Cecal microbial analysis showed that, compared with the TN group, the abundance of *Merdimonas*, *Proteobacteria*, and *Galbibacter* significantly increased (*p* < 0.05), while the abundance of *Bacteroides*, *Prevotella*, and *Parasutterella* significantly declined (*p* < 0.05). Furthermore, the NA-supplemented group exhibited a significant rise in the enrichment of *Olivibacter* and *Flintibacter* (*p* < 0.05) and a marked reduction in the enrichment of *Proteobacteria* (*p* < 0.05). Additionally, the addition of NA significantly elevated the levels of acetic acid, butyrate, and propionic acid in the cecum (*p* < 0.05). In conclusion, dietary NA supplementation mitigated the adverse effects of HS on TBsf, primarily by enriching beneficial microbiota such as *Bacteroides* and *Flintibacter*, and promoting the production of SCFAs like butyric acid and acetic acid.

## 1 Introduction

Meteorological stressors, particularly HS, pose a critical challenge to poultry production sustainability in subtropical regions. Broiler chickens exhibit heightened thermal sensitivity due to their genetically selected traits for rapid growth, high feed conversion efficiency, and physiological limitations in thermoregulation, including the absence of functional sweat glands and dense feather coverage. These unique physiological constraints make broiler chickens particularly vulnerable to HS (1, 2). Numerous studies have indicated that HS can elevate cloacal temperature, increase respiratory rate and heart rate, decrease appetite, and reduce feed intake in broiler chickens. These alterations in physiological functions have significant negative impacts on production performance (3, 4). Recent studies have revealed a complex microbial ecosystem in the caecum, where the microbiota and its metabolic by-products significantly enhance nutrient breakdown and absorption, drive metabolic processes, and boost overall growth efficiency in poultry (5). The caecum hosts a dense microbial community, and the composition, density, and metabolic by-products of the caecal microbiota are key factors in regulating intestinal function. Numerous studies have demonstrated that heat stress disrupts bacterial density and metabolic profiles in the caeca of broiler chickens (6, 7). Thus, augmenting gut microbiota or identifying microbial modifiers may provide a basis for mitigating heat stress-induced disturbances in the gut microbiota that lead to impaired performance. Heat stress can induce physiological and metabolic disorders as well as abnormal nutrient metabolism in broiler chickens, thereby reducing systemic immunity and exerting highly detrimental effects on broiler production. Consequently, discovering effective strategies to alleviate heat stress in broilers and preserve their production performance presents a significant challenge that needs to be addressed.

To date, various intervention strategies have been proposed to alleviate the adverse effects of HS on poultry. These approaches mainly include optimizing environmental conditions and feeding systems, incorporating appropriate dietary supplements, and breeding birds with improved heat tolerance. A recent development in this field is the creation of heat-resistant chickens through embryonic manipulation (2). Among these strategies, incorporating appropriate heat stress—alleviating dietary additives is deemed a highly effective method to mitigate the adverse effects of heat stress on broiler production (8). Heat stress-resistant additives, which primarily include vitamins and minerals, oligomannose, probiotics, resveratrol, betaine, and others, have been crucial in maintaining vital organ function, enhancing immune responses, improving growth performance, sustaining intestinal microbial balance, and boosting antioxidant capacity in broilers when supplemented during heat stress periods (9, 10).

Nicotinic acid functions as a component of several essential coenzymes that play a crucial role in energy metabolism (11, 12). Studies have demonstrated that NA supplementation can mitigate heat stress under high-temperature conditions by inducing vasodilation (13). This vasodilation enhances heat dissipation, establishing a thermal gradient that increases peripheral heat loss and consequently reduces core body temperature (12, 14).

An expanding body of research indicates that NA may mitigate the detrimental impact of HS on livestock productivity. However, the relationship between NA, cecal microbial communities, their fermentation products, and the production performance of TBsf under HS remains underexplored. Consequently, this study was designed to examine the effects of NA on growth performance and heat stress-induced alterations in intestinal microbiota in TBsf. Different concentrations of NA were added to the diet to determine its influence, providing a theoretical basis for the development of heat stress mitigation strategies in avian species.

## 2 Materials and methods

### 2.1 Ethics statement

Animal husbandry and experiments were conducted in accordance with the Chinese Animal Welfare Guidelines approved by the Jiangxi Agricultural University Animal Care and Use Committee (loan number JXAULL-2021 1,213).

### 2.2 Design and management

A total of 150 male TBsf, 180 days old and averaging 2802.00 ± 226.80 g in weight, were sourced from a nearby farm. These birds were then randomly allocated into five distinct treatment cohorts, with 30 chickens per group. Temperature and humidity were controlled using electric heaters and humidifiers in each room: including TN, with room temperature controlled at 24–25°C and humidity at 45–55%, and HS with temperature controlled at 31–33°C and humidity at 70–75% and supplemented with different levels of NA in HS + NA. The TN and HS cohorts consumed standard diets. In contrast, the HS + NA group received a standard diet supplemented with NA. The NA was initially mixed with a segment of the feed and subsequently diluted and blended thrice to yield three distinct feed portions. Each portion contained varying concentrations of NA: 200, 400, and 800 mg/kg. These groups were designated as HS + NA_0.02_ (200 mg/kg NA), HS + NA_0.04_ (400 mg/kg NA), and HS + NA_0.08_ (800 mg/kg NA), respectively. Nutrient levels in the diet adhered to the 1994 National Research Council guidelines. The composition and nutrient content of the basal diet are detailed in Table 1. Birds had unrestricted access to both food and water. Rooms were fitted with thermostatically controlled heaters and fans to ensure proper air circulation. An artificial light source was maintained on continuously to provide illumination throughout the experiment. The study encompassed a total duration of 35 days, which included an initial 7-day pre-feeding stage followed by a 28-day primary experimental phase. Utilizing a cyclic heat stress model, the regimen commenced heating at 8:30 daily. By 9:00, conditions met the heat stress criteria. Cooling initiated at 17:00 to maintain consistent temperatures between the HS and TN phases.

**Table 1:** Composition and nutrient levels of the basal diet (air-dry basis).

Items	
Ingredients (%)	
Corn	67.60
Soybean meal	22.00
Fish meal	2.00
Soybean oil	4.00
Dicalcium phosphate	1.00
Limestone	1.10
_DL_-Methionine	0.09
Threonine	0.05
Salt	0.30
Zeolite powder	1.36
Premix^*^	0.50
Total	100.00
Nutrient levels (%)	
Metabolizable energy (MJ/kg)	12.97
Crude protein	16.19
Calcium	0.81
Available phosphorus	0.38
Threonine	0.69
Lysine	0.86
Methionine	0.36
Methionine + cystine	0.65

Provided per kilogram of diet: vitamin A 11250 IU, vitamin D3 3,750 IU, vitamin E 30 IU, vitamin K 1.75 mg, thiamin 2.75 mg, riboflavin 9.30 mg, calcium pantothenate 18.75, niacin 37.50, vitamin B6 3.75 mg, biotin 0.15 mg, folic acid 1.80 mg, vitamin B12 0.028 mg, choline chloride 720 mg, Fe (from ferrous sulfate) 80 mg, Cu (from cupric chloride) 8 mg, Mn (from manganous sulfate) 80 mg, Zn (from Zinc sulfate) 60 mg, I (from calcium iodate) 0.35 mg, Se (from sodium selenite) 0.15 mg.

### 2.3 Production performance

Chicken body weights were measured after a 24-h fast, and daily feed intake for each replicate was recorded at 08:00 on both the first and last days of the 28-day trial. The average daily feed intake (ADFI), ADG, and F: G were subsequently calculated.

### 2.4 Nutrients and temperature–humidity index (THI)

During the experiment, feed samples were collected. Feces were gathered from each cage over the last 3 days. The collected feces were weighed and homogenized, after which two 150 g samples were extracted. One sample was treated with 10% H₂SO₄ for the analysis of crude protein (CP) content. The samples were subsequently analyzed for dry matter (DM), CP, and ether extract (EE) using the methods prescribed by AOAC International (2005) (15). The CP content was measured using a Kjeldahl nitrogen determinator (model KDN-19C), while the EE content was determined using a Soxhlet extractor (provided by Guanyin Biotechnology).

Temperature and humidity levels were assessed using a thermometer, with readings taken every hour from 9:00. to 17:00. The formula refers to the study of Zou et al. (12).

### 2.5 Body temperature and HSP70 level

On days 7, 14, 21, and 28 of the heat stress period, the cloacal temperature of each chicken was measured using a digital thermometer. At the conclusion of the heat stress period (day 28), five chickens were randomly selected from each dietary and temperature group. Blood samples were collected from the wing veins to obtain serum. The levels of HSP70 in the serum samples were assessed using a specific kit purchased from the Institute of Bioengineering in Nanjing.

### 2.6 Cecal contents analyses

Upon completion of the experiment and subsequent slaughter, approximately 2 g of cecal contents from each chicken were collected into lyophilization tubes. The samples were initially flash-frozen in liquid nitrogen and subsequently transferred to a − 80°C freezer for storage prior to microbiological and volatile fatty acid analyses.

### 2.7 SCFAs

The levels of volatile fatty acids in the cecal contents were quantified using a GC-8860 gas chromatograph (Agilent Technologies 7820A, USA), following previously documented methodologies (16). Samples weighing precisely 0.5 g were thoroughly dispersed in 3 mL of distilled water by vigorous agitation for 1 min, then centrifuged for 10 min. The resulting supernatant was combined with a 20% metaphosphoric acid solution and centrifuged again for 10 min. One milliliter of this supernatant was vigorously mixed with 2 mL of ethyl acetate, allowed to stand for 5 min, and prepared for analysis. The injection volume was 1.0 μL, with helium gas as the carrier gas. The column temperature was initially set at 110°C and then gradually increased to 200°C. The injector and detector temperatures were set at 250°C and 350°C, respectively.

### 2.8 16S rRNA

DNA extraction was performed using the E. Z. N. A. Soil DNA Kit from Omega Bio-tek, Inc., USA. DNA quality was assessed by 1% agarose gel electrophoresis and quantified using a Nanodrop 2000 spectrophotometer (ThermoFisher Scientific, Inc., USA). The V3-V4 hypervariable region was amplified with universal primers in the ABI 9700 PCR system (Applied Biosystems, Inc., USA). The target amplicon size of the PCR products was confirmed by 1% agarose gel electrophoresis. The purification of the PCR products was then automated using the Agencourt AMPure XP system (Beckman Coulter, Inc., USA).

### 2.9 Illumina Miseq sequencing

This approach utilized the Illumina Miseq/Novaseq 6,000 platform (Illumina, Inc., USA) with a PE250/PE300 sequencing strategy. The original sequence data have been deposited in the NCBI Sequence Read Archive (SRA) under accession number PRJNA1128340.

### 2.10 Statistical analysis

T-tests for independent samples were utilized to contrast the TN and HS scenarios. In addition, a one-way ANOVA was applied within the HS context, and the entirety of the statistical evaluations was carried out with SPSS 23.0 software. Significance was attributed to outcomes where *p* < 0.05, and high significance was recognized when *p* < 0.01.

## 3 Results

### 3.1 THI and anal temperature

**Figure 1:**
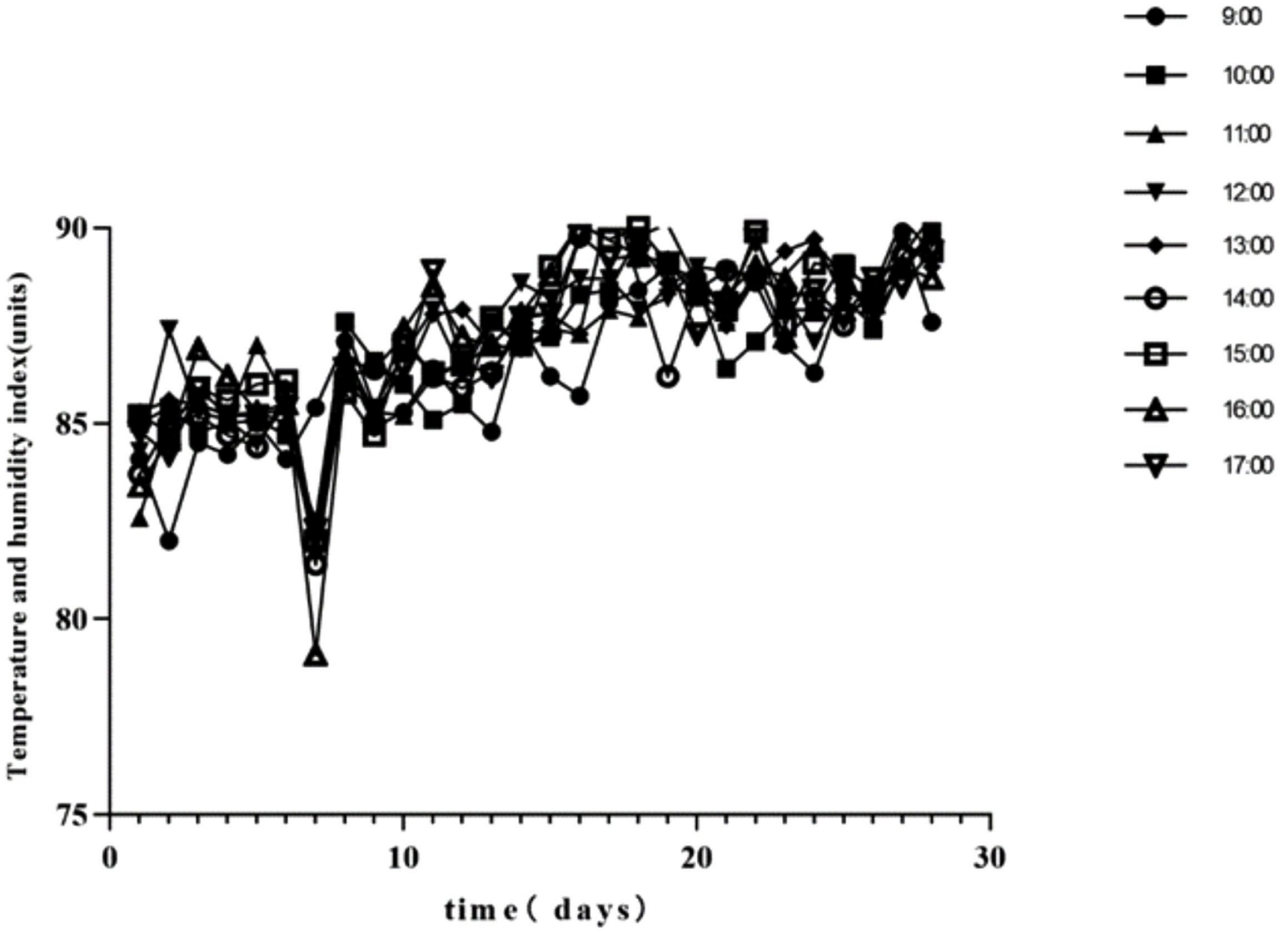
Daily changes in the temperature and humidity index (THI) at different times during the experimental period.

Throughout the experiment, the mean daily THI values for the HS treatments exceeded 79. The daily variation of hourly THI values from 09:00 to 17:00 is depicted in Figure 1. Table 2 summarizes the findings regarding body temperature. On days 7, 14, 21, and 28, chickens in the HS group exhibited significantly higher body temperatures compared to the control group. The HS + NA_0.08_ group showed a marked reduction in body temperature on days 7, 14, and 21 relative to the HS group (*p* < 0.05).

**Table 2:** Effect of niacin on body temperature (°C) of Taihe black-boned silky fowl under heat stress.

Items	TN	HS	HS + NA_0.02_	HS + NA_0.04_	HS + NA_0.08_	*P*_1_	SEM	*P*_2_
7 days	42.46	42.94^a^	42.66^ab^	42.64^ab^	42.56^bc^	0.014	0.12	0.026
14 days	42.54	43.00^A^	42.82^A^	42.74^AB^	42.46^BC^	0.037	0.12	0.003
21 days	42.12	43.04^a^	42.88^ab^	42.76^ab^	42.62^bc^	0.008	0.15	0.049
28 days	42.36	43.02	42.82	42.76	42.70	0.005	0.12	0.072

TN: the basal diet, thermoneutral condition; HS, heat stress-treated group; HS+NA_0.02_, HS+NA_0.04_, HS+NA_0.08_ heat stress plus niacin-treated group. SEM, standard error of the mean. P_1_ is the *p*-value of the *t*-test for TN and HS groups, and P_2_ is the *p*-value of one-way ANOVA for the four groups that received heat stress treatments. Between the four groups that received heat stress treatments, peer data with the same letter on the shoulder or no letter indicate insignificant differences (*p* > 0.05), different lowercase letters indicate significant differences (*p* < 0.05), and different uppercase letters indicate highly significant differences (*p* < 0.01).

### 3.2 Growth performance

Table 3 demonstrates that HS significantly reduced ADG and ADFI to a highly significant extent (*p* < 0.01), while also significantly increasing the F/G compared to the TN group (*p* < 0.05). In contrast, in TBsf supplemented with NA, ADG and ADFI increased, and the F/G ratio significantly decreased (*p* < 0.05). Notably, the most pronounced effects were observed in the HS + NA_0.08_ group.

**Table 3:** Effect of dietary niacin addition on Taihe black-boned silky fowl production performance under heat stress.

Items	TN	HS	HS + NA_0.02_	HS + NA_0.04_	HS + NA_0.08_	*P_1_*	SEM	*P_2_*
ADFI (g)	320.24	228.32^a^	238.62^ab^	235.04^ab^	255.88^bc^	<0.001	8.95	0.042
ADG (g)	16.71	1.93^A^	2.57^AB^	3.86^BC^	4.57^C^	<0.001	0.59	0.002
F/G (g/g)	19.54	134.98^a^	100.36^ab^	68.12^b^	57.42^b^	0.002	23.65	0.02

TN: the basal diet, thermoneutral condition; HS, heat stress treated group; HS+NA_0.02_, HS+NA_0.04_, HS+NA_0.08_ heat stress plus niacin treated group. SEM, standard error of the mean. P_1_ is the *p*-value of the *t*-test for TN and HS groups, and P_2_ is the *p*-value of one-way ANOVA for the four groups that received heat stress treatments. Between the four groups that received heat stress treatments, peer data with the same letter on the shoulder or no letter indicate insignificant differences (*p* > 0.05), different lowercase letters indicate significant differences (*p* < 0.05), and different uppercase letters indicate highly significant differences (*p* < 0.01). ADFI, average daily feed intake; ADG, average daily gain; F/G, feed-to-gain ratio.

### 3.3 Nutrient apparent digestibility

Table 4 demonstrates that HS significantly decreased the apparent digestibility of CP, achieving statistical significance (*p* < 0.05). The addition of NA resulted in a numerical improvement in nutrient digestibility compared to the HS group, although this did not reach statistical significance (*p* > 0.05).

**Table 4:** Effects of dietary niacin on nutrient apparent digestibility of Taihe black-boned silky fowl under heat stress (%).

Items	TN	HS	HS + NA_0.02_	HS + NA_0.04_	HS + NA_0.08_	*P*_1_	SEM	*P*_2_
DM	81.90	81.38	82.09	82.74	81.84	0.566	0.61	0.197
CP	56.30	51.72	51.90	51.56	50.92	0.038	1.74	0.947
EE	87.6	87.36	87.65	86.30	87.67	0.669	1.22	0.648

TN: the basal diet, thermoneutral condition; SEM, standard error of the mean. P_1_ is the *p*-value of the *t*-test for TN and HS groups, and P_2_ is the *p*-value of ANOVA for the four groups that received heat stress treatments. Between the four groups that received heat stress treatments, peer data with the same letter on the shoulder or no letter indicate insignificant differences (*p* > 0.05), different lowercase letters indicate significant differences (*p* < 0.05), and different uppercase letters indicate highly significant differences (*p* < 0.01). ME, dry matter; EE, ether extract; CP, crude proteins.

### 3.4 HSP70 in serum

**Figure 2:**
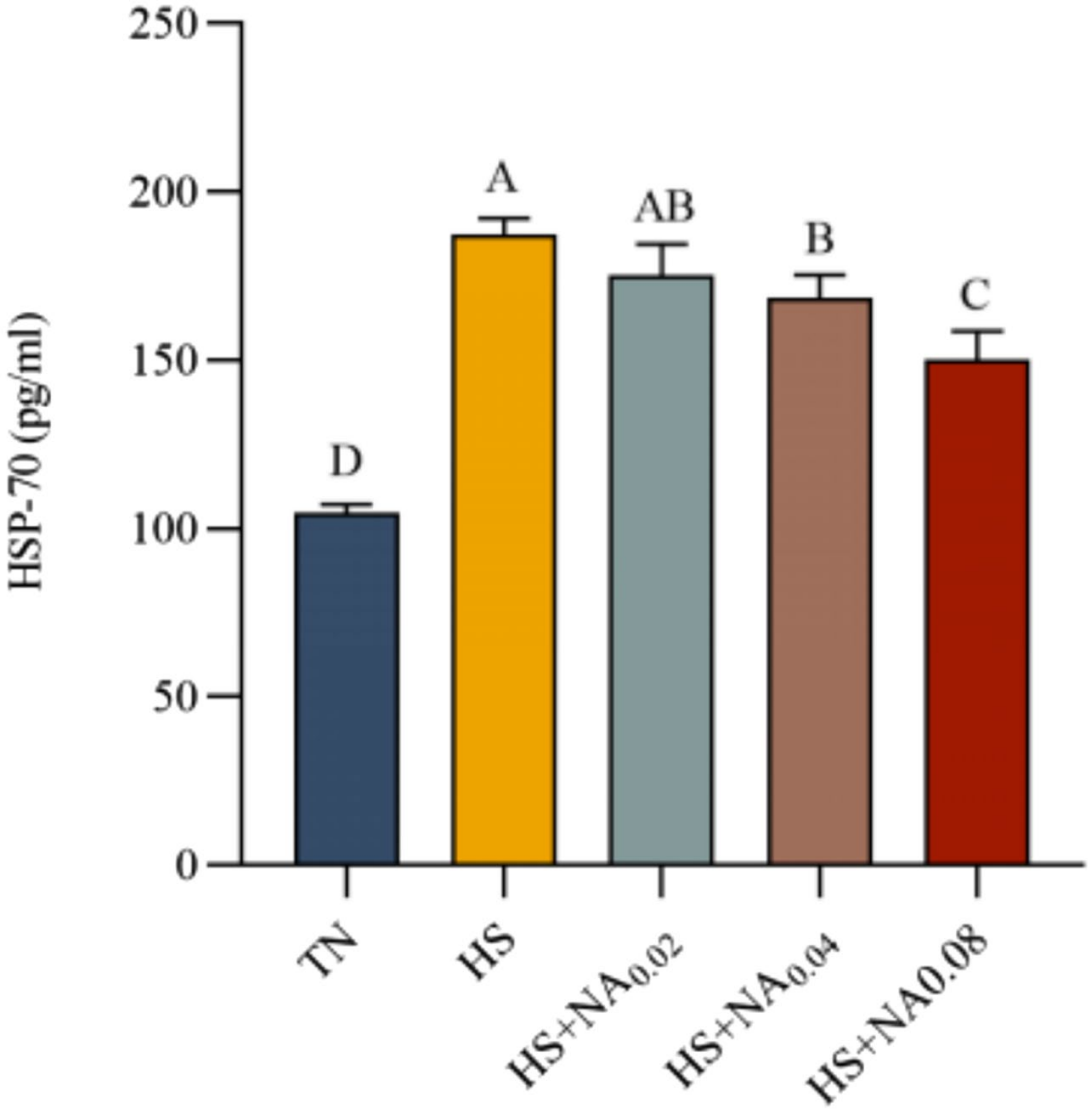
Effect of dietary niacin supplementation on HSP70 content in the serum of Taihe black-boned silky fowl under heat stress. TN: the basal diet, thermoneutral condition (*n* = 5); HS, heat stress treated group (*n* = 5); HS + NA_0.02_, HS + NA_0.04_, HS + NA_0.08_ heat stress plus niacin treated group (*n* = 5).

Figure 2 shows that heat stress significantly increased serum HSP70 concentrations in TBsf (*p* < 0.001). Compared with the HS group, the HS + NA_0.04_ and HS + NA_0.08_ groups exhibited significantly lower serum HSP70 levels (*p* < 0.05). Although the HS + NA_0.02_ group had reduced HSP70 expression compared with the HS group, the difference was not statistically significant (*p* > 0.05).

### 3.5 Diversity of microorganisms in the cecum

Within the scope of this study, 25 samples yielded a cumulative total of 567,725 valid sequences. The community’s richness and *α*-diversity were assessed utilizing the Shannon, Simpson, and Chao1 indices, with the results presented in Table 5. Under consistent sequencing depth, the α-diversity metrics of Shannon, Simpson, and Chao1 indices exhibited no significant variance across the five groups (*p* > 0.05).

**Table 5:** Effect of dietary niacin on microbial diversity in the cecum of Taihe black-boned silky fowl under heat stress.

Items	TN	HS	HS + NA_0.02_	HS + NA_0.04_	HS + NA_0.08_	*P*_1_	SEM	*P*_2_
Simpson	0.98	0.98	0.97	0.98	0.96	1.000	0.01	0.365
Shannon	8.16	8.31	7.85	8.33	7.63	0.681	0.41	0.274
Chao1	3270.44	3535.65	3310.53	3410.11	3096.08	0.259	229.19	0.315

TN: the basal diet, thermoneutral condition; HS, heat stress treated group; HS+NA_0.02_, HS+NA_0.04_, HS+NA_0.08_ heat stress plus niacin treated group. SEM, standard error of the mean. P_1_ is the *p*-value of the *t*-test for TN and HS groups, and P_2_ is the *p*-value of one-way ANOVA for the four groups that received heat stress treatments. Between the four groups that received heat stress treatments, peer data with the same letter on the shoulder or no letter indicate insignificant differences (*p* > 0.05), different lowercase letters indicate significant differences (*p* < 0.05), and different uppercase letters indicate highly significant differences (*p* < 0.01).

**Figure 3:**
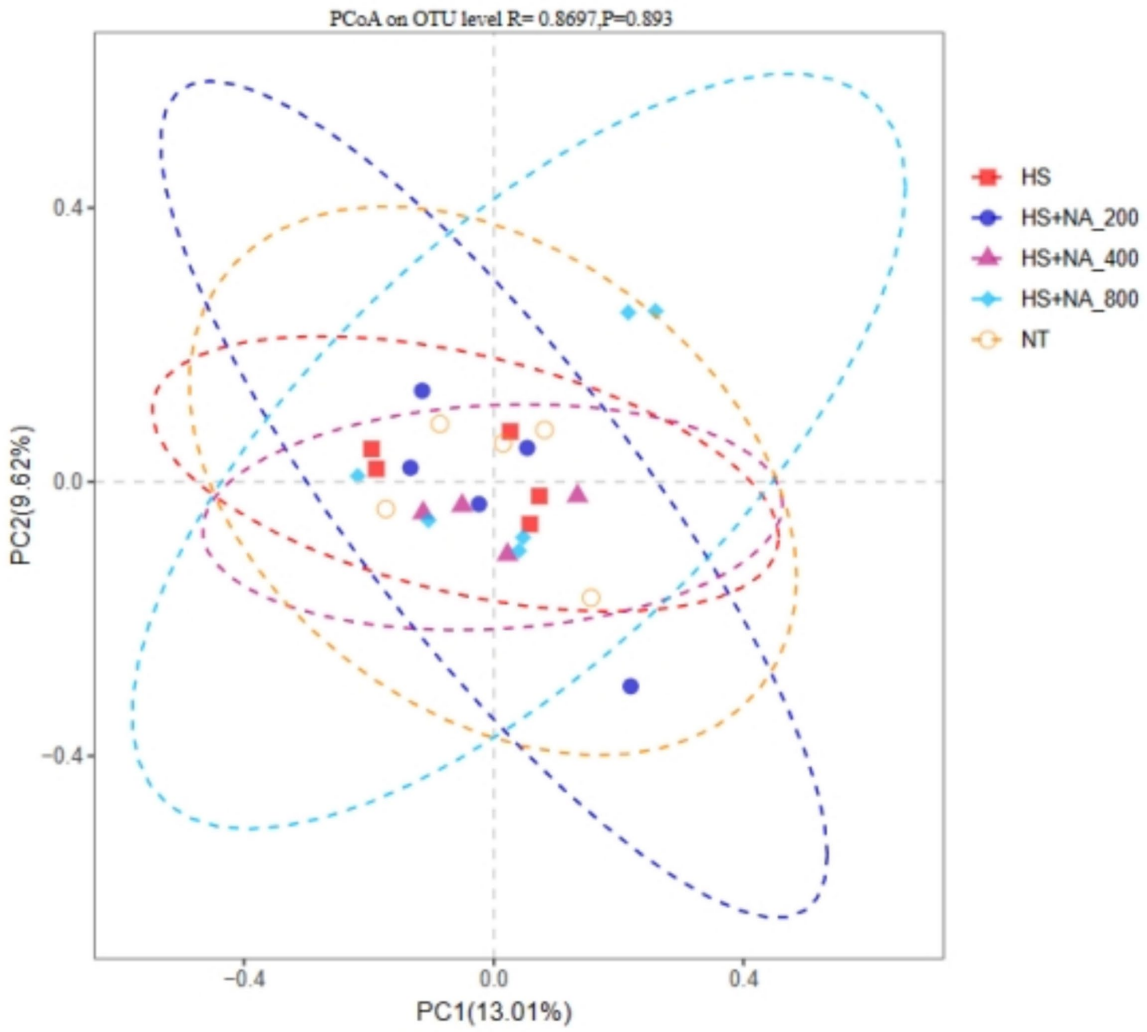
Principal coordinate analysis (PCoA) plot based on OTU abundance. TN: the basal diet, thermoneutral condition (*n* = 5); HS, heat stress treated group (*n* = 5); HS + NA_0.02_, HS + NA_0.04_, HS + NA_0.08_ heat stress plus niacin treated group (*n* = 5).

To evaluate the similarity between flora, *β* diversity was assessed using weighted standardized UniFrac. Figure 3 shows no significant variation in cecal microbiota among the groups (*p* = 0.8697).

**Figure 4:**
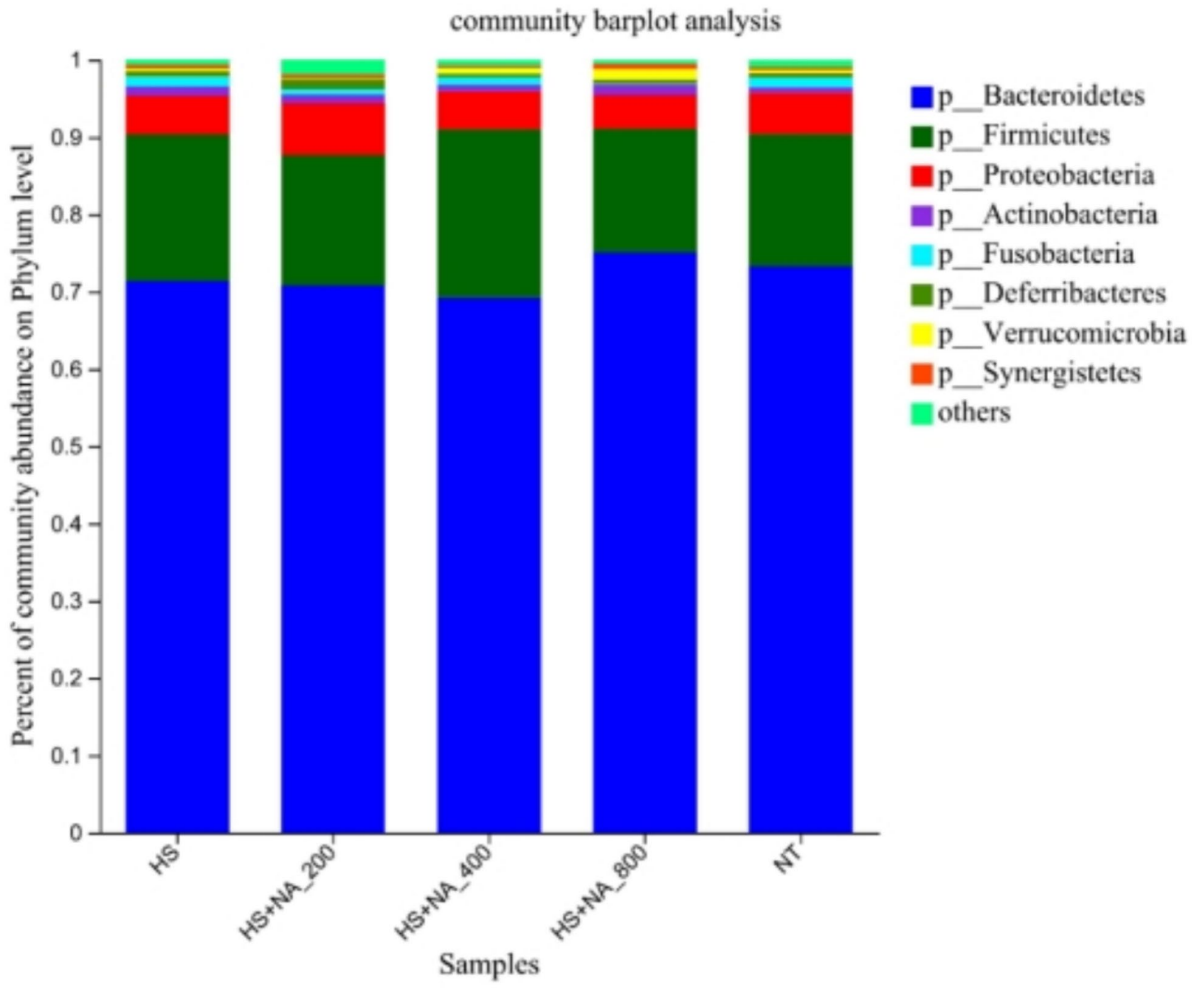
Relative abundance distribution of cecal flora at the phylum level. TN: the basal diet, thermoneutral condition (*n* = 5); HS, heat stress treated group (*n* = 5); HS + NA0.02, HS + NA0.04, HS + NA0.08 heat stress plus niacin treated group (*n* = 5).

**Figure 5:**
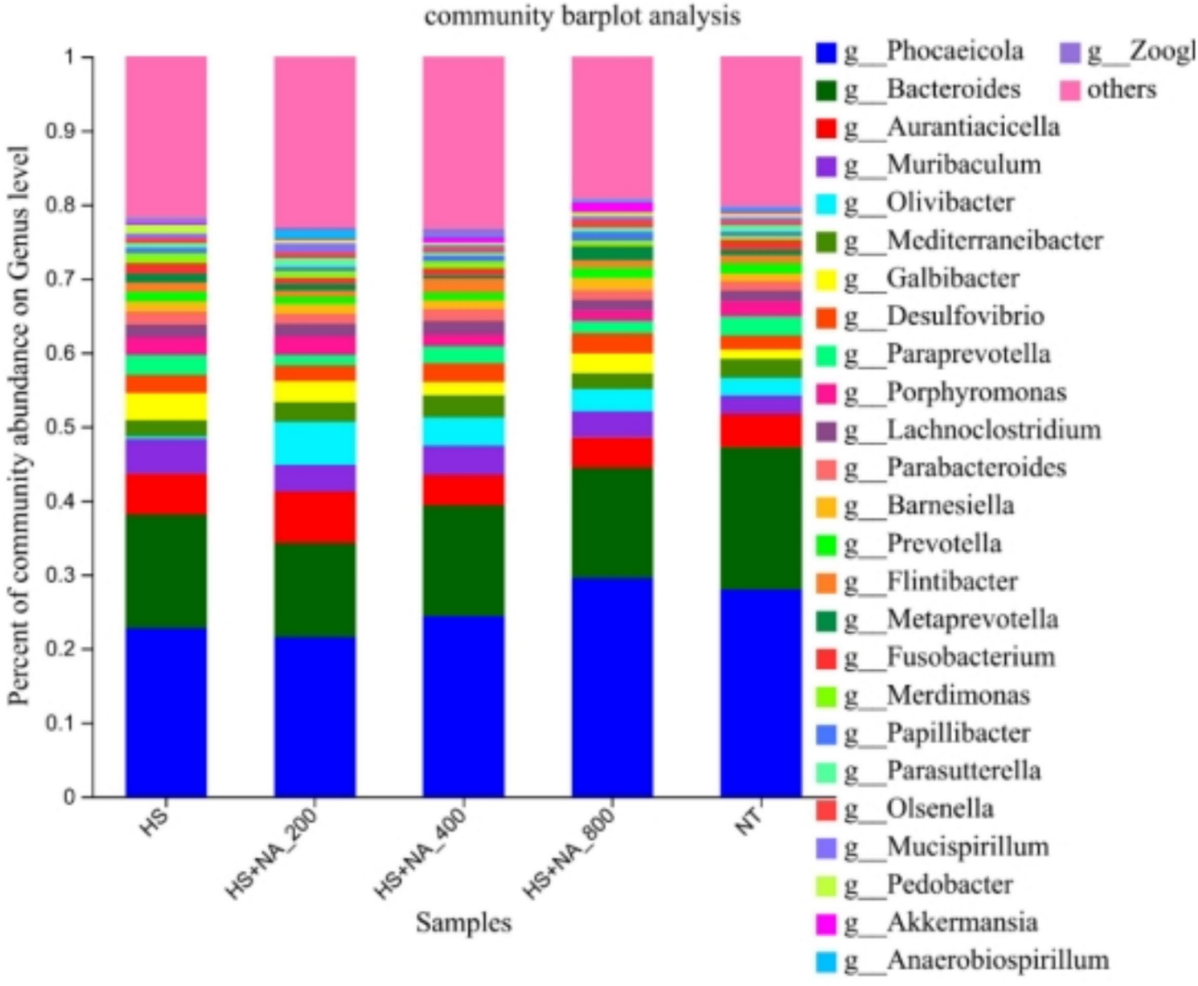
Relative abundance distribution of cecal flora at the genus level. TN: the basal diet, thermoneutral condition (*n* = 5); HS, heat stress treated group (*n* = 5); HS + NA_0.02_, HS + NA_0.04_, HS + NA_0.08_ heat stress plus niacin treated group (*n* = 5).

This inquiry evaluates the consequences of HS on the cecal microflora’s structure and abundance in TBsf, as well as the modulatory influence of NA, across both phylum and genus taxonomic levels. Specifically, the analysis was conducted at the phylum level (see Table 6 and Figure 4) and at the genus level (see Table 7 and Figure 5). The eight most abundant phyla identified were *Bacteroidetes, Firmicutes, Proteobacteria, Actinobacteria, Fusobacteria, Deferribacteria, Verrucomicrobia,* and *Synergistetes*, as illustrated in Figure 4. The enrichment of Proteobacteria was significantly lower in the HS compared to the TN (*p* < 0.05). Significantly, NA supplementation resulted in a reduced enrichment of Proteobacteria, with the HS + NA_0.04_ group showing the most notable impact (*p* < 0.05). Supplementation with NA markedly decreased the enrichment of Proteobacteria, with the HS + NA0.08 group yielding the most positive results (*p* < 0.05).

**Table 6:** Effect of niacin on the structure of the bacterial flora of the cecum (phylum level) %.

Items	TN	HS	HS + NA_0.02_	HS + NA_0.04_	HS + NA_0.08_	*P*_1_	SEM	*P*_2_
*p__Bacteroidetes*	76.23	69.58^a^	71.09^ab^	72.57^ab^	79.86^b^	0.132	2.91	0.032
*p__Firmicutes*	19.68	17.01	18.28	23.77	18.45	0.401	3.25	0.234
*p__Proteobacteria*	3.8	6.11^a^	5.26^ab^	3.39^b^	3.54^b^	0.014	0.8	0.023
*p__Actinobacteria*	0.7	1.11	1.12	0.84	1.37	0.272	0.43	0.678
*p__Fusobacteria*	1.28	1.38	0.76	0.97	0.13	0.943	0.84	0.53
*p__Deferribacteres*	0.55	0.59	1.19	0.44	0.44	0.926	0.49	0.399
*p__Verrucomicrobia*	0.44	0.37	0.2	0.76	1.42	0.887	0.8	0.463
*p__Synergistetes*	0.38	0.43	0.4	0.3	0.69	0.856	0.26	0.505

TN: the basal diet, thermoneutral condition; HS, heat stress treated group; HS+NA_0.02_, HS+NA_0.04_, HS+NA_0.08_ heat stress plus niacin treated group. SEM, standard error of the mean. P_1_ is the *p*-value of the *t*-test for TN and HS groups, and P_2_ is the *p*-value of one-way ANOVA for the four groups that received heat stress treatments. Between the four groups that received heat stress treatments, peer data with the same letter on the shoulder or no letter indicate insignificant differences (*p* > 0.05), different lowercase letters indicate significant differences (*p* < 0.05), and different uppercase letters indicate highly significant differences (*p* < 0.01).

**Table 7:** Effect of niacin on the structure of the bacterial flora of the cecum (genus level) %.

Items	TN	HS	HS + NA_0.02_	HS + NA_0.04_	HS + NA_0.08_	*P*_1_	SEM	*P*_2_
*g__Phocaeicola*	28	22.72	21.56	24.38	29.57	0.166	5.07	0.433
*g__Bacteroides*	21.69	14.91	13.39	16.89	16.2	0.027	1.81	0.3
*g__Aurantiacicella*	4.49	5.52	6.96	4.12	4.14	0.529	2.06	0.477
*g__Muribaculum*	3.18	2.92a	4.57b	4.79b	4.04ab	0.824	1.06	0.089
*g__Olivibacter*	0.74	0.11A	0.20A	2.36B	0.52A	0.156	0.56	0.006
*g__Mediterraneibacter*	2.55	2.21	2.66	2.99	2.09	0.709	0.84	0.688
*g__Galbibacter*	1.35	4.41	1.99	2.05	3.13	0.026	0.96	0.072
*g__Desulfovibrio*	1.65	2.98	1.54	1.9	1.82	0.148	0.54	0.109
*g__Paraprevotella*	3.77	1.95	1.92	3.4	2.21	0.31	0.66	0.159
*g__Porphyromonas*	1.6	3.16	1.78	1.33	1.11	0.116	0.71	0.078
*g__Lachnoclostridium*	1.37	1.71	1.6	1.8	1.48	0.289	0.25	0.592
*g__Parabacteroides*	1.3	1.763	1.4	1.62	1.32	0.225	0.36	0.654
*g__Barnesiella*	1.08	1.41	1.34	1.2	1.61	0.449	0.43	0.812
*g__Prevotella*	1.7	0.82	1.19	1.29	1.71	0.022	0.3	0.1
*g__Flintibacter*	1.55	0.74a	1.11a	2.26b	1.46b	0.051	0.34	0.011
*g__Metaprevotella*	1.25	0.46	1.55	0.62	2.79	0.678	0.9	0.106
*g__Fusobacterium*	1.26	1.38	0.76	0.97	0.13	0.898	0.84	0.53
*g__Merdimonas*	0.49	1.64	0.81	0.82	0.58	0.03	0.36	0.055
*g__Papillibacter*	0.59	0.72	0.56	0.77	1.13	0.718	0.57	0.785
*g__Parasutterella*	1.2	0.54	1.66	0.46	0.72	0.029	0.11	0.15

TN: the basal diet, thermoneutral condition; HS, heat stress treated group; HS+NA_0.02_, HS+NA_0.04_, HS+NA_0.08_ heat stress plus niacin treated group. SEM, standard error of the mean. P_1_ is the *p*-value of the *t*-test for TN and HS groups, and P_2_ is the *p*-value of one-way ANOVA for the four groups that received heat stress treatments. Between the four groups that received heat stress treatments, peer data with the same letter on the shoulder or no letter indicate insignificant differences (*p* > 0.05), different lowercase letters indicate significant differences (*p* < 0.05), and different uppercase letters indicate highly significant differences (*p* < 0.01).

In Table 7, the HS group exhibited significantly elevated levels of *Galbibacter* and *Merdimonas* (*p* < 0.05), while the levels of *Bacteroides*, *Prevotella*, and *Parasutterella* were notably reduced (*p* < 0.05). Furthermore, the HS + NA_0.04_ exhibited a rise in the level of *Olivibacter*, and both the HS + NA_0.04_ and HS + NA_0.08_ groups demonstrated heightened relative abundances of *Flintibacter,* relative to the HS (*p* < 0.05).

### 3.6 SCFAs

Table 8 reveals that the levels of propionic and butyric acids were significantly reduced under HS conditions (*p* < 0.001). Supplementation of the diet with NA led to a substantial and highly significant increase in cecal propionic and butyrate levels compared to the HS (*p* < 0.001). Additionally, the HS + NA_0.08_ exhibited a significant elevation in cecal acetic acid content when compared to the HS (*p* < 0.05).

**Table 8:** Effect of niacin on the concentration of volatile fatty acids in the cecum of Taihe black-boned silky fowl kens under heat stress.

Items	TN	HS	HS + NA_0.02_	HS + NA_0.04_	HS + NA_0.08_	*P*_1_	SEM	*P*_2_
Acetic acid mmol/L	58.36	51.58^a^	54.59^a^	55.80^a^	68.36^b^	0.295	5.66	0.043
Propionic acid mmol/L	14.25	12.35^A^	13.95^B^	15.26^C^	18.57^D^	<0.001	0.36	<0.001
Butyric acid mmol/L	7.74	3.04^A^	7.99^B^	5.32^B^	8.86^BC^	<0.001	0.29	<0.001

TN: the basal diet, thermoneutral condition; HS, heat stress treated group; HS+NA_0.02_, HS+NA_0.04_, HS+NA_0.08_ heat stress plus niacin treated group. SEM, standard error of the mean. P_1_ is the *p*-value of the *t*-test for TN and HS groups, and P_2_ is the *p*-value of one-way ANOVA for the four groups that received heat stress treatments. Between the four groups that received heat stress treatments, peer data with the same letter on the shoulder or no letter indicate insignificant differences (*p* > 0.05), different lowercase letters indicate significant differences (*p* < 0.05), and different uppercase letters indicate highly significant differences (*p* < 0.01).

## 4 Discussion

The ambient temperature and relative humidity are the primary determinants of heat exchange on the body surface of livestock. The THI serves as a prevalent measure for assessing the degree of HS in livestock (12). During the study, the THI values for the HS and HS + NA treatments surpassed 79 for a total of 28 days, indicating that the TBsf were exposed to high or severe stress. This confirms the effective induction of heat stress throughout the experimental period. HSP70 is widely recognized as a biomarker of elevated body temperature in response to heat stress (17, 18). During this study, the body temperature in the TBsf subjected to HS was markedly elevated compared to that under thermoneutral conditions throughout the experiment. Conversely, this parameter was notably reduced following NA supplementation. This effect could be attributed to NA’s capacity to induce vasodilation and enhance the rate of blood flow to the skin surface under HS (12). Thus, the addition of NA can reduce skin temperature, possibly by inducing vasodilation and accelerating blood flow, thereby alleviating HS.

Previous experimental evidence has consistently demonstrated that HS negatively impacts the growth performance of livestock via multiple pathways. These include alterations in appetite, decreased feed consumption, compromised intestinal nutrient uptake, and reductions in both gastrointestinal motility and the activity of digestive enzymes (19, 20). Numerous studies indicate that heat stress diminishes ADFI and ADG (21). Incorporating NA into the diet notably enhanced ADG and reduced the F/G relative to the HS group. These findings align with prior research (12, 22). Studies have shown that supplementing livestock diets with NA improves feed nitrogen utilization, enhances biological and metabolic functions, and increases nutrient uptake. These effects are attributed not only to NA’s ability to facilitate protein absorption but also to its role in promoting the growth of beneficial cecal bacteria and inhibiting detrimental bacteria, thereby contributing to the maintenance of intestinal health (23).

In monogastric animals, the cecum is a key site for converting dietary fiber into essential nutrients, including volatile fatty acids and microbial proteins (24). The cecum hosts a complex microbial ecosystem, and maintaining a stable microbiota is crucial for enhancing feed utilization, optimizing animal performance, bolstering immune function, preserving gut barrier integrity, and promoting overall animal health. Moreover, the cecal microbiota provides the host with energy through the fermentation of indigestible carbohydrates, producing SCFAs and ammonia (25). In the present study, we observed an increase in the abundance and diversity of gut microbiota at the phylum level. The primary phyla detected were *Bacteroidetes*, *Firmicutes*, *Proteobacteria*, and *Actinobacteria*, which collectively accounted for approximately 96% of the total microbiota. Notably, *Firmicutes* are potentially linked to the host’s energy acquisition and contribute to material and energy metabolism (26). In our study, the HS + NA_0.08_ group significantly elevated the level of *Bacteroidetes*. Concurrently, the HS + NA_0.02_ and HS + NA_0.08_ groups significantly reduced the relative abundance of *Proteobacteria*. Organisms belonging to the phylum *Bacteroidetes* are implicated in the breakdown of polysaccharides and proteins, which aids in nutrient absorption and is instrumental in sustaining intestinal homeostasis and health (27, 28). *Proteobacteria* is frequently associated with various disorders, including inflammatory bowel disease (IBD), and is acknowledged as a phylum that may be harmful to intestinal health (29).

Research has demonstrated that NA can modulate the diversity of rumen microbiota, stimulate the synthesis of microbial proteins, and augment the concentration of SCFAs in the rumen (30, 31). Additionally, previous studies have shown that NA improves the structure of the microflora and increases SCFA concentrations in the piglet colon (32). SCFAs, primarily generated through the fermentation of dietary fiber by gut microbiota, include acetate, propionate, and butyrate. These fatty acids are involved in carbohydrate and lipid metabolism and can modulate the endocrine system, potentially correlating positively with host weight gain (33). In this study, the nicotinic acid (NA)-supplemented group exhibited a higher relative abundance of *Muribaculum*, *Olivibacter*, *Prevotella*, *Flintibacter*, and *Parasutterella* at the genus level. *Muribaculum* and *Parasutterella* belong to the Lactobacillaceae family. The metabolites of Lactobacillus, primarily SCFAs with a notable emphasis on butyrate, are consistent with the findings of this study. The research demonstrated that NA dietary supplementation significantly increased the butyrate concentration in cecal contents. Butyrate is recognized for its significant contribution to warding off pathogenic infections and mitigating intestinal inflammation (34). During NA treatment, a significant rise in the prevalence of *Muribaculum* and *Parasutterella* was detected. This finding corroborates earlier research regarding the levels of these bacterial species. Such an increase can result in elevated butyrate levels in the intestine, fortify the intestinal barrier, and assist in mitigating the inflammatory response within the colon (35). Moreover, in this study, the NA-treated group exhibited increased levels of *Prevotella* and *Flintibacter*. Notably, the fermentation products of these bacteria primarily consisted of butyrate (36, 37). *Bacteroides* produce metabolites, predominantly SCFAs such as acetate, which have been recognized for their significant role in mitigating intestinal inflammation (38). Concurrently, acetate is believed to prompt the secretion of growth hormone-releasing peptide, thereby enhancing the release of growth hormone (39). The secretion of growth hormone-releasing peptide is considered a beneficial factor for enhancing pig production performance and augmenting body weight gain (40). These findings are consistent with the results of the NA group treatments in this study, which demonstrated an increased relative abundance of Bacteroides and a higher ADG. Furthermore, SCFAs influence appetite and modulate feed intake in livestock (41), a result that aligns with the present research findings. In this research, the NA-treated group exhibited significantly higher average daily feed intake (ADFI) and acetate levels.

**Figure 6:**
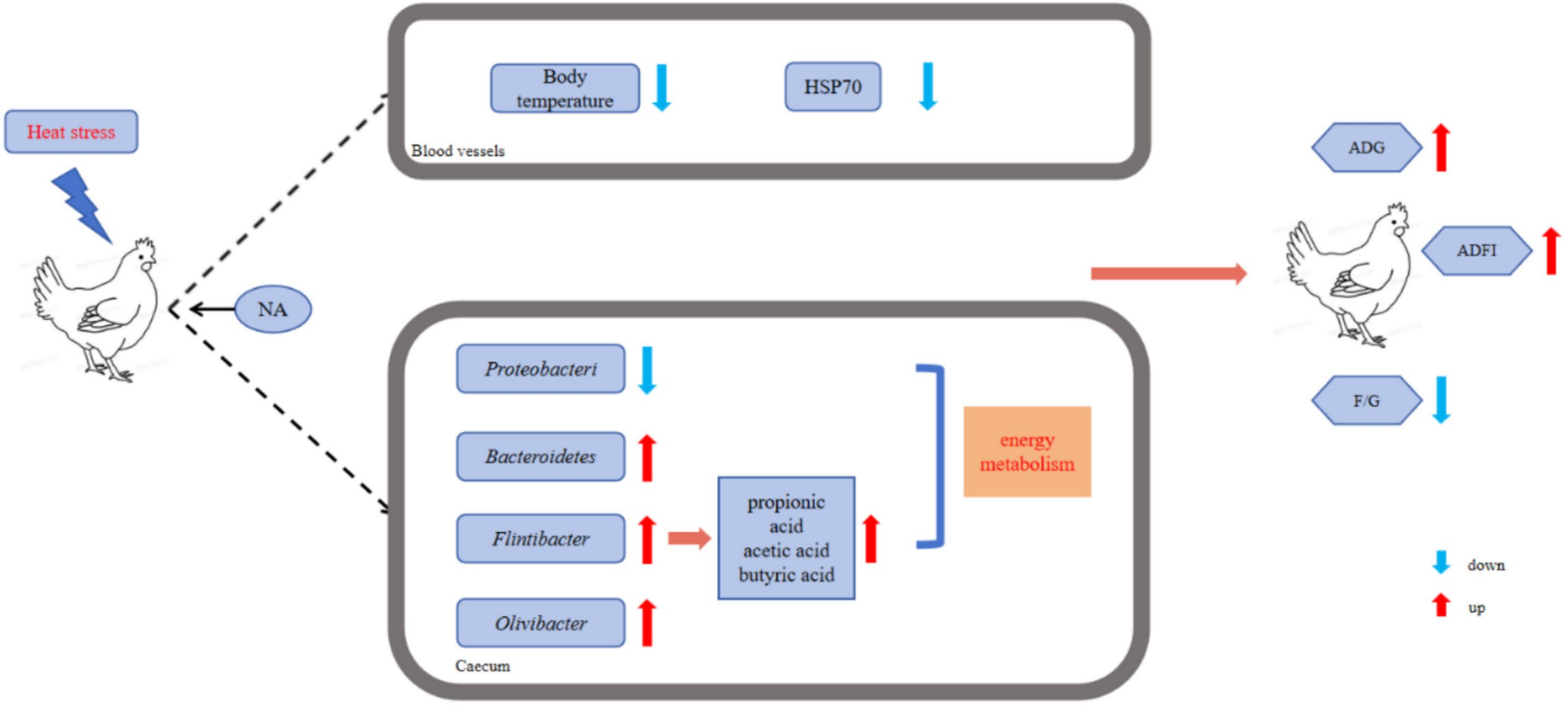
Schematic diagram illustrating the mechanism by which NA protects Taihe black-boned silky fowl from HS induced decline in productive performance.

Here, NA dietary supplementation resulted in reduced relative abundance of the genus-level bacteria *Porphyromonas* and *Flintibacter*, contrasting with the HS group. Prior research has associated *Porphyromonas gingivalis* with ulcerative colitis (UC). Peptidylarginine deiminase (PPAD), produced by *Porphyromonas gingivalis*, is recognized as a virulence factor that can trigger an inflammatory response (42). As depicted in Figure 6, the addition of NA reduces the number of pathogenic bacteria, promotes the proliferation of beneficial bacteria, and modulates the composition and concentration of volatile fatty acids in the cecum. These changes enhance nutrient digestion and absorption, ultimately leading to improved animal performance.

## 5 Conclusion

In conclusion, the addition of NA can mitigate the adverse impacts of HS and enhance, to a certain degree, the relative abundance of cecal microorganisms as well as production performance. This research provides a theoretical foundation for NA’s alleviating effects on heat-stressed TBsf. Consequently, NA is anticipated to serve as a preventative and control additive for heat stress in TBsf, with an optimal dosage ranging from 400 to 800 mg/kg.

## Data Availability

The datasets presented in this study can be found in online repositories. The names of the repository/repositories and accession number(s) can be found at: https://www.ncbi.nlm.nih.gov/, PRJNA1128340.
